# The Principles and Practice of Modern Surgery

**Published:** 1842-08-06

**Authors:** 


*The Principles and Practice of Modern Surgery.* By ROBERT DRUITT. From the Second London Edition. Illustrated with 50 Wood Engravings. With Notes and Comments by JOSHUA B. FLINT, M. D., M. M. S. S. Lecturer on Therapeutic and Operative Surgery in the Louisville Academy of Medicine, &c. Philadelphia, 1842. pp. 534.

This work but little merits the title bestowed on it by its cisatlantic sponsor; nor do we know that the original one of "the Surgeon's Vade Mecum" was any better deserved. It is professedly a compilation, but an unskilful one, with but little harmony in the heterogeneous materials—ancient exploded dogmas and modern crudities being placed in no enviable juxtaposition. Our author shows but small practical acquaintance with his subject—little study, and less patient thought and reflection. In a laudatory preface he tells us that the present edition contains, he believes, "every novelty in surgery that deserves mention." This self-complacency excites a legitimate smile, and we are sorry to disturb it, but when Mr. Druitt thus openly challenges criticism, we can assure him that his work is as much characterized by sins of omission as by those of commission. An occasional marginal reference to the periodicals of the day is but a poor substitute for a clear condensed statement of the substance of the best current opinions; and this can only be accomplished by one who has really an intimate acquaintance with the principles and practice of modern surgery, derived from study and observation. Mr. Druitt, on the contrary, frequently displays an unwarrantable ignorance of the actual condition of Surgical Science in Europe.

The notes of Prof. FLINT are judicious and well written, and make us regret that the modesty of this accomplished surgeon prevented his supplying more frequently the deficiencies of the text. We are sorry to differ with him in regard to the actual merits of Mr. Druitt's book.

Dr. FLINT states, that nothing in his whole surgical experience in the West has been more remarkable than the disproportioned frequency of diseases of the rectum and the adjacent textures. He thinks that this disposition is principally to be attributed to the indiscriminate use of drastic purgatives, both as remedials as well as prophylactics. He represents the practice of a large proportion of the practitioners of the Valley of the Mississippi to consist almost entirely in the exhibition of violent cathartics; and this simple and easy empiricism is imitated by the patients themselves whenever they are ill. The mischievous practice here deprecated, as well as the mercurial mania, have unfortunately a much wider range than the "far West," and are but too general throughout the country.

The Doctor thinks, and justly so, that, independent of these immediate evils, another necessarily results—the encouragement which these pernicious doctrines give to the various quack pills, which are everywhere vended in such quantities, and do such irremediable injury.

